# Diagnostic Salivary Tests for SARS-CoV-2

**DOI:** 10.1177/0022034520969670

**Published:** 2020-10-31

**Authors:** L. Azzi, V. Maurino, A. Baj, M. Dani, A. d’Aiuto, M. Fasano, M. Lualdi, F. Sessa, T. Alberio

**Affiliations:** 1 Unit of Oral Medicine and Pathology, ASST dei Sette Laghi–Ospedale di Circolo e Fondazione Macchi, Department of Medicine and Surgery, University of Insubria, Varese, Italy; 2 Laboratory of Clinical Microbiology, ASST dei Sette Laghi–Ospedale di Circolo e Fondazione Macchi, Department of Medicine and Surgery, University of Insubria, Varese, Italy; 3 Laboratory of Biochemistry and Functional Proteomics, Department of Science and High Technology, Busto Arsizio (VA), Italy; 4 Unit of Pathology, ASST dei Sette Laghi–Ospedale di Circolo e Fondazione Macchi, Department of Medicine and Surgery, University of Insubria, Varese, Italy

**Keywords:** COVID-19, SARS-CoV-2, coronavirus, Severe acute respiratory syndrome-related coronavirus, saliva, point-of-care testing

## Abstract

The diagnosis of Severe Acute Respiratory Syndrome Coronavirus 2 (SARS-CoV-2) infection relies on the detection of viral RNA by real-time reverse transcription polymerase chain reaction (rRT-PCR) performed with respiratory specimens, especially nasopharyngeal swabs. However, this procedure requires specialized medical personnel, centralized laboratory facilities, and time to provide results (from several hours up to 1 d). In addition, there is a non-negligible risk of viral transmission for the operator who performs the procedure. For these reasons, several studies have suggested the use of other body fluids, including saliva, for the detection of SARS-CoV-2. The use of saliva as a diagnostic specimen has numerous advantages: it is easily self-collected by the patient with almost no discomfort, it does not require specialized health care personnel for its management, and it reduces the risks for the operator. In the past few months, several scientific papers, media, and companies have announced the development of new salivary tests to detect SARS-CoV-2 infection. Posterior oropharyngeal saliva should be distinguished from oral saliva, since the former is a part of respiratory secretions, while the latter is produced by the salivary glands, which are outside the respiratory tract. Saliva can be analyzed through standard (rRT-PCR) or rapid molecular biology tests (direct rRT-PCR without extraction), although, in a hospital setting, these procedures may be performed only in addition to nasopharyngeal swabs to minimize the incidence of false-negative results. Conversely, the promising role of saliva in the diagnosis of SARS-CoV-2 infection is highlighted by the emergence of point-of-care technologies and, most important, point-of-need devices. Indeed, these devices can be directly used in workplaces, airports, schools, cinemas, and shopping centers. An example is the recently described Rapid Salivary Test, an antigen test based on the lateral flow assay, which detects the presence of the virus by identifying the spike protein in the saliva within a few minutes.

## Introduction

Ten months have passed since the Chinese health authorities informed the World Health Organization (WHO) about the outbreak of a novel coronavirus-associated pneumonia in the province of Hubei and the city of Wuhan (Zhu, Zhang, et al. 2020). This novel coronavirus was soon named Severe Acute Respiratory Syndrome Coronavirus 2 (SARS-CoV-2) because of its close relationship to the virus responsible for the 2003 SARS epidemic (SARS-CoV). The disease caused by this new infectious agent was called Coronavirus Disease 2019 (COVID-19). Despite the fact that SARS-CoV-2 shares 80% sequence similarity with SARS-CoV, its contagiousness appears to be much higher, as demonstrated by the worldwide diffusion of the infection, with over 40,000,000 cases officially diagnosed and 1,128,000 deaths by the end of October 2020 (World Health Organization 2020).

At the start of the pandemic, a diagnostic protocol was recommended by the WHO. Based on the experience from other respiratory infectious diseases, including SARS in 2003, detection of viral RNA by real-time reverse transcription polymerase chain reaction (rRT-PCR) in respiratory specimens was recognized as the reference standard for the diagnosis of SARS-CoV-2 infection (Corman et al. 2020). Among different respiratory specimens, the nasopharyngeal swab (NPS) was recommended as the first choice for testing in terms of sensitivity.

However, this technique entails the main limitation of requiring several hours up to 1 d to generate results, thus reducing the possibility of rapid diagnoses made directly on the field and its deployment in a mass screening program. During the peak of the COVID-19 epidemic, the crowding of centers designated to analyze the specimens caused interruption of many other diagnostic procedures, which had a major impact on the delivery of essential health services for chronic illnesses. Furthermore, the collection of respiratory specimens requires specialized health care personnel and is associated with a nonnegligible risk of viral transmission. The procedure itself may be associated with pharynx irritation, sneezing, and cough, increasing the risk for the operator who is in contact with the patient (Ng et al. 2020). Finally, the sensitivity of testing using this specimen may vary significantly depending on the interval between exposure and the sampling procedure (Wiersinga et al. 2020).

For these reasons, several studies have suggested detection of SARS-CoV-2 using other body fluids such as urine, stool, tears, and saliva (Sun et al. 2020). Among these body fluids, saliva has attracted both scientific attention and public approval. It is now regarded as an alternative or complementary sample to the nasopharyngeal swab. As a proof of this, the Food and Drug Administration (FDA) recently approved the Emergency Use Authorization of several saliva-based tests, such as those proposed by Rutgers’ RUCDR Infinite Biologics and Yale School of Public Health. This stance is consistent with the finding that salivary droplets represent the main source of human-to-human transmission of SARS-CoV-2 infection (Han and Ivanovski 2020). The use of saliva as a diagnostic specimen offers numerous advantages: it is easily self-collected by the patient with almost no discomfort, it does not require specialized health care personnel for its management, and it reduces the risks for the operator. As a result, in the past few months, several scientific papers, media, and companies have reported the development of new salivary tests to detect SARS-CoV-2 infection. The aim of this review is to provide an update on this topic, synthesizing the latest research and comparing the different methods and techniques developed for the salivary diagnosis of COVID-19.

## The Detection of SARS-CoV-2 in Saliva

The idea that saliva droplets could represent an important source of infection and a suitable sample for diagnosis was highlighted in 2003 during the SARS outbreak (Wang et al. 2004). Analogous considerations were made for the Middle Eastern respiratory syndrome coronavirus (MERS-CoV) outbreak (Adhikari et al. 2019). Similarly, the eruption of the new pandemic and its severe course have drawn the attention of researchers to these issues. Within the family of coronaviruses, SARS-CoV-2 has the highest basic reproductive rate (R_0_). Indeed, the viral load for SARS-CoV peaks 6 to 11 d after the symptom onset, while the load for SARS-CoV-2 peaks at the onset of symptoms and then declines. This feature highlights the role of presymptomatic individuals in the transmission of the infection (Petersen et al. 2020), as well as the role of asymptomatic people (Lavezzo et al. 2020).

### Posterior Oropharyngeal Saliva

**Figure 1.**
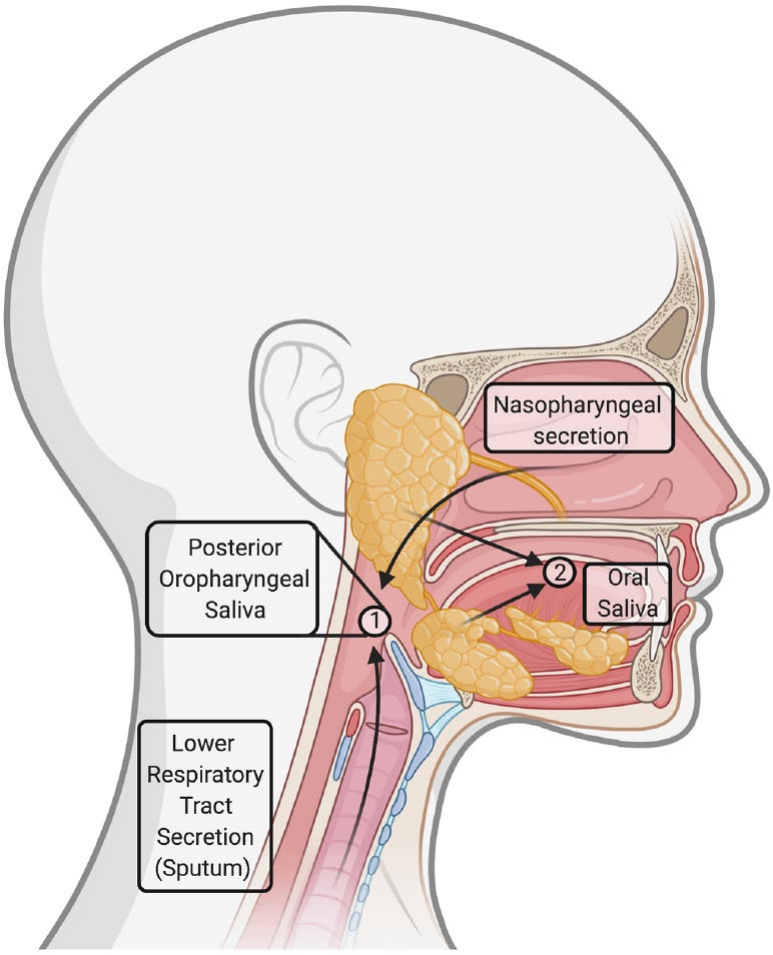
Different salivary samples. Posterior oropharyngeal saliva is the secretion produced when coughing or clearing one’s throat, and it belongs to the respiratory secretions, admixing secretions from both the upper (nasopharynx) and lower (bronchi, lungs) airways (number 1 in the circle). In contrast, oral saliva is produced by the salivary glands and does not belong to the group of respiratory specimens (number 2 in the circle). However, a clear distinction between these 2 kinds of samples is not feasible and does not fall within the aim of laboratory clinical diagnosis of Coronavirus Disease 2019. The saliva produced when coughing will contain oral saliva, while a small quantity of oropharyngeal secretions may be present in oral saliva.

Detection of SARS-CoV-2 in the saliva by rRT-PCR was originally described by To and coworkers (To, Tsang, Yip, et al. 2020). In their study, the authors analyzed 23 COVID-19 patients with different severities of illness and reported that 87% of them had detectable viral RNA in their saliva (To, Tsang, Leung, et al. 2020). This group has also previously underlined the role played by saliva in the diagnosis of respiratory infections, such as those caused by influenza or other coronaviruses (To et al. 2017). The saliva collected in these studies was defined as *posterior oropharyngeal saliva*. This means that the patient expectorates pharyngeal secretions, which belong to respiratory secretions, and not only oral saliva produced by the salivary glands, which are outside the respiratory tract (Fig. 1).

The use of posterior oropharyngeal saliva as a specimen to detect SARS-CoV-2 has also been described in other studies, which emphasized the fact that such samples might contain both bronchopulmonary and nasopharyngeal secretions. Notably, these studies were conducted in the Hong Kong Special Administrative Region, where health authorities conducted a surveillance campaign by collecting posterior oropharyngeal samples at locations such as airports.

### Oral Saliva

Our group was the first to report the detection of SARS-CoV-2 in oral saliva by rRT-PCR in April 2020 (Azzi, Carcano, Gianfagna, et al. 2020). However, in our study, we recruited only hospitalized COVID-19 patients affected by a severe form of the disease. In the following weeks, other studies investigated the role of saliva as a diagnostic tool by also recruiting symptomatic patients with a milder form of the disease (Becker et al. 2020; Caulley et al. 2020; Iwasaki et al. 2020; Jamal et al. 2020; Kim, Lee, et al. 2020; McCormick-Baw et al. 2020; Migueres et al. 2020; Nagura-Ikeda et al. 2020; Pasomsub et al. 2020; Williams et al. 2020; Wyllie et al. 2020). Most of these studies reported the results of analyses conducted with small- and medium-sized patient cohorts (i.e., 200 subjects or fewer), although studies with larger cohorts (i.e., about 1,000 subjects) have been recently published (Caulley et al. 2020; Zhu, Guo, et al. 2020).

Remarkably, several of these studies reported positive salivary samples concurrently with negative NPSs. The reasons underlying this finding remain unclear and could be related to several factors, including incorrect performance of the NPS procedure or different patterns of the viral and clinical course of the infection. The published data suggest that a combination of salivary and respiratory specimens in a hospital setting may increase the overall sensitivity and reduce the number of false-negative results.

Nevertheless, it is worrisome that more than one report, including one from our group, showed that some COVID-19 patients may have a negative NPS while their salivary sample is and remains positive when tested by rRT-PCR (Azzi, Carcano, Dalla Gasperina, et al. 2020). This finding raises the question of whether all the patients who show 2 consecutive negative tests with NPSs are actually not contagious.

**Figure 2.**
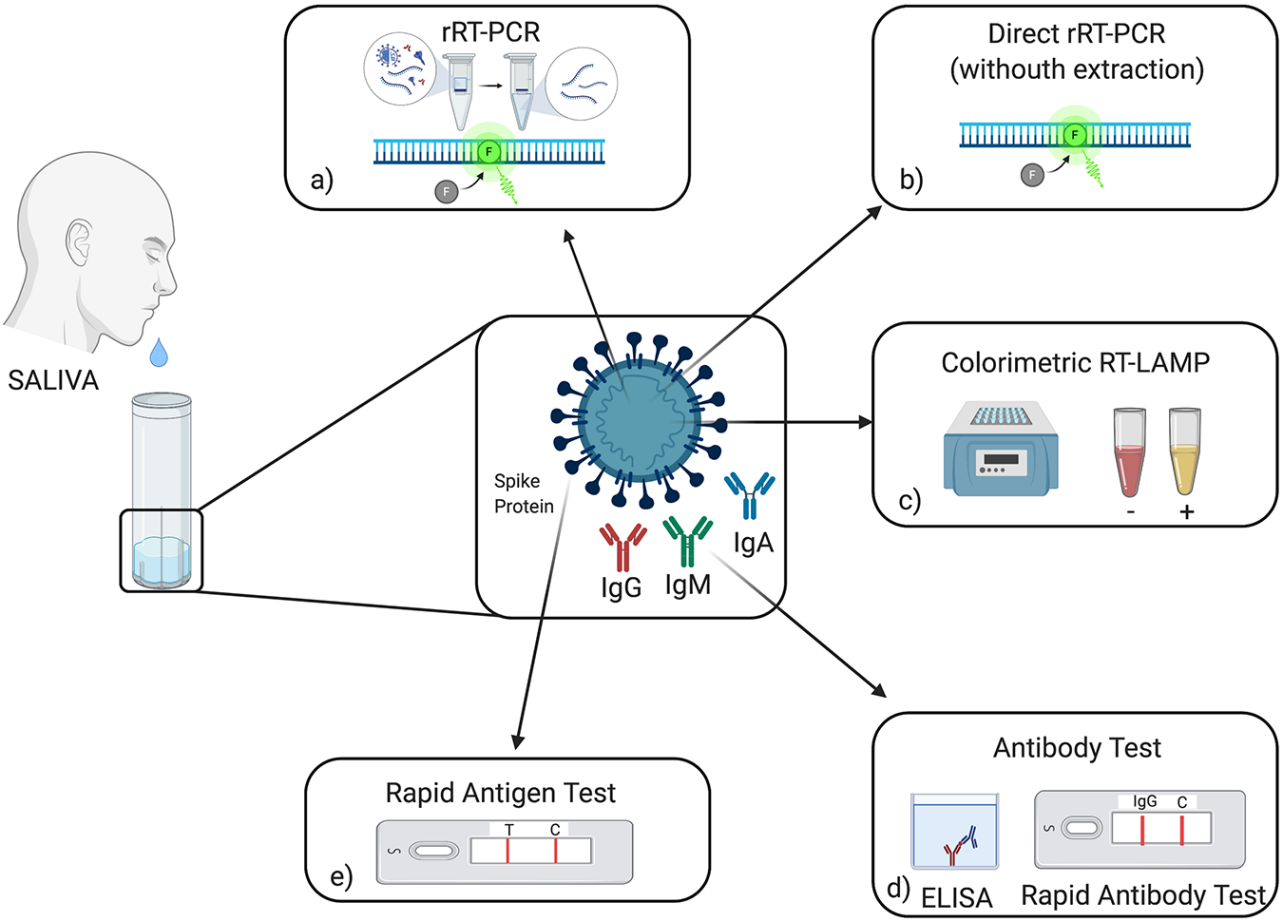
Coronavirus Disease 2019 (COVID-19) salivary diagnosis procedures. Saliva is collected with the drooling technique, avoiding coughing or expectoration. (**a**) Real-time reverse transcription polymerase chain reaction (rRT-PCR): this test represents the reference standard for COVID-19 diagnosis and is usually performed on respiratory specimens, but it can be used on saliva. (**b**) Direct rRT-PCR allows quicker diagnosis because RNA isolation is avoided. (**c**) Colorimetric reverse transcription loop-mediated isothermal amplification (RT-LAMP) is a point-of-care technology that allows rapid detection of viral RNA by combining LAMP technology with a colorimetric assay. (**d**) Antibody detection in saliva can be performed both with enzyme-linked immunosorbent assay (ELISA) or lateral flow assay. (**e**) Rapid salivary test is an antigen test based on the lateral flow assay, which shows great promise for mass screening.

Figure 2 summarizes the different diagnostic methods that can be used for detecting SARS-CoV-2 in saliva.

## Molecular-Based Tests for the Detection of SARS-CoV-2 in Saliva

Molecular-based diagnostics inform clinicians of the presence of SARS-CoV-2 by identifying its genomic material, that is, the viral RNA, in the analyzed sample. These procedures represent the reference standard for the diagnosis of viral infections. They require dedicated equipment, that is, thermal cyclers, expensive reagents, specialized personnel, and laboratory infrastructures. Therefore, they are suitable within the context of a hospital or a tertiary referral center.

### rRT-PCR

In the SARS-CoV-2 outbreak, the use of rRT-PCR as the reference standard diagnostic procedure was drawn from the experience gained with SARS-CoV in 2003. The diagnostic strategy encompasses the use of rRT-PCR assays performing the nucleic acid amplification test (NAAT) by targeting 1 or more genes in the SARS-CoV-2 genome. This procedure typically consists of RNA isolation, purification, reverse transcription to complementary DNA (cDNA), amplification, detection, and quantification by the incorporation of a fluorescent probe.

**Figure 3.**
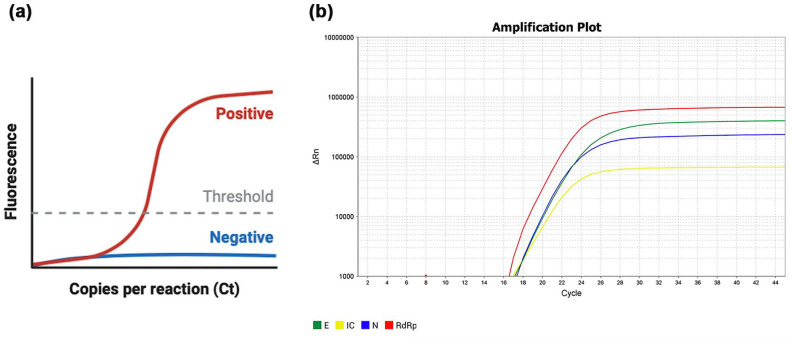
Real-time reverse transcription polymerase chain reaction (rRT-PCR) of a salivary positive sample. (**a**) Schematic illustration of an rRT-PCR result. The amplification curve for positive samples follows a sigmoid trend (i.e., the relative fluorescence intensity increases, with an exponential middle tract, until a plateau phase). No increase in fluorescence is observed when the sample is negative. The threshold is placed so to intersect the amplification curves at the beginning of the exponential tract. The cycle threshold (Ct) represents the cycle number at which the amplification curve intersects the threshold line and is an indicator of the quantity of the amplified target gene. The lower the Ct value, the higher the amount of the target gene and then the viral load. (**b**) An example of amplification curves in *log* scale for a salivary sample that tested positive for the presence of all 3 genes associated with SARS-CoV-2 (*E, N*, and *RdRp*). The internal control (IC), whose viral load is known, is used as comparison to quantify the viral load of the sample.

A validated protocol endorsed by the WHO entails a first-line screening assay with amplification of the *envelope* (*E*) gene, followed by a confirmatory assay with amplification of the RNA-dependent RNA polymerase (RdRp) region of the *Orf1b* gene, and then an additional potential confirmatory assay by amplification of the *nucleocapsid* (*N*) gene (Fig. 3). Another recognized protocol has been proposed by the US Centers for Disease Control and Prevention (CDC) and encompasses the use of 2019-nCoV N1 and N2 primer-probes sets along with the *RNAse* *P* gene as an internal control. These procedures are described as techniques with the highest sensitivity in viral RNA detection. However, they have shown several limitations for deployment in mass screening programs since the beginning of the pandemic (Lippi, Simundic, et al. 2020). The most important limitation is the time required for the diagnosis (several hours up to 1 d) and the crowding of centers designated to analyze specimens.

Consequently, some companies have developed new diagnostic testing solutions such as a more rapid PCR assay, which allows faster assessment of the infection in central facilities dedicated to COVID-19 diagnosis (Bordi et al. 2020). These methods allow more rapid diagnosis by direct rRT-PCR without RNA extraction. Similarly, other companies have developed rRT-PCR devices that include fully automated commercial systems that can shorten the bench time per sample by nearly 90%, reducing the possibility of mistakes during specimen handling and allowing analysis of a larger number of patients in a shorter time frame (Chen et al. 2020; Lippi, Mattiuzzi, et al. 2020; Nagura-Ikeda et al. 2020).

Another strategy that has been recently introduced to address the reduced resources in low-prevalence areas is sample pooling. The saliva pool of either 5 of 10 samples allows the detection of viral RNA in the pool, and further individual sample testing is performed only in pools that tested positive by rRT-PCR (Watkins et al. 2020).

#### Diagnostic Accuracy of Salivary rRT-PCR

In a group of studies, the detection of viral RNA in saliva was compared with that of nasopharyngeal and/or oropharyngeal swabs (OPSs) performed on the same day of the salivary collection (Table).

**Table.** Diagnostic Accuracy Values of Real-Time Reverse Transcription Polymerase Chain Reaction (rRT-PCR) Salivary Analysis Reported in the Literature.

Study	Date	Cohort	Number	Respiratory Sample	Target Genes	Sensitivity, n; %	Specificity, n; %	Positive Predictive Value, %	Negative Predictive Value, %	Notes
Williams et al.	2020, April	Ambulatory patients, screening	522	NPS	ORF1a, ORF8	33/39; 84.62%	49/50; 98%	97.06	89.09	
Becker et al.	2020, May	1) Symptomatic individuals (CDC criteria); 2) Convalescent subjects	8824	NPS	S, N, ORF1ab (RdRp)	40-60%20-50%	97-100%75-94%	n/a	n/a	Different collection kits, probes, and laboratories
McCormick-Baw et al.	2020, May	Emergency Department and COVID-19 hospitalized patients (not severe)	156	NPS	E and N2	47/49; 95.92%	105/106; 99.06%	97.92	98.13	
Pasomsub et al.	2020, May	Symptomatic individuals	200	NPS and OPS	N, ORF1ab	16/19; 84.21%	179/181; 98.9%	88.9^a^	98.4	
Iwasaki et al.	2020, June	Suspicious subjects and COVID-19 patients (mild-moderate)	76 (10 + 66)	NPS	Taqman probe 2019-nCoV 2.9.1 Japan	8/9; 88.89%	66/67; 98.51%	88.89	98.51	
Jamal et al.	2020, June	COVID-19 hospitalized patients	91	NPS	E, N, RdRp	44/64; 68.75%	19/27; 70.37%	69.84	48.72	Frozen samples
Zhu et al.	2020, June	12 independent cohorts (various degrees)	944	NPS or OPS	not specified	382/442; 86.43%	487/502; 97.01%	96.22	89.03	
Nagura-Ikeda et al.	2020, July	Laboratory-confirmed COVID-19	103 (88 symptomatic and 15 asymptomatic)	NPS or OPS	N1 and N2	84/103; 81.6%	n/a	n/a	n/a	Frozen samples; respiratory swabs and salivary collection not at the same day
Caulley et al.	2020, August	High risk asymptomatic and mildly symptomatic individuals	1939	NPS or OPS	E gene	34/56; 60.71%	1869/1883; 99.26%	70.83	98.84	Viricidal fluid in the collection kit; 2 laboratories
Kim et al.	2020, August	COVID-19 hospitalized patients (asymptomatic and symptomatic, various degrees)	15^b^	NPS or OPS	E, RdRp	n/a	n/a	n/a	n/a	
Migueres et al.	2020, August	Hospitalized and ambulatory patients (symptomatic and asymptomatic)	123	NPS	RdRp	34/41; 82.93%	79/82; 96.34%	91.89	91.86	
Wyllie et al.	2020, August	COVID-19 hospitalized patients (severe); asymptomatic health care workers	70495	NPS	N1 and N2	n/a	n/a	n/a	n/a	Sensitivity of saliva 1-5 days: 81% Sensitivity of NPS 1-5 days: 71%

CDC, Centers for Disease Control and Prevention; COVID-19, Coronavirus Disease 2019; n/a, not applicable; NPS, nasopharyngeal swab; OPS, oropharyngeal/throat swab; RdRp, RNA-dependent RNA polymerase.

a The 2 “false positives” later reported anosmia.

b Evaluation on more than 1 sample per patient; only without sputum.

Some of these studies compared the sensitivity of both the salivary and respiratory samples in detecting the presence of SARS-CoV-2 infection in the analyzed patients. The results were heterogeneous, with saliva showing a lower diagnostic accuracy than the NPS/OPS (i.e., saliva: 55% to 72% vs. NPS/OPS: 82% to 98%) in some cases. In other cases, the values for saliva were equal to or even higher than those recorded with the NPS/OPS (i.e., saliva: 82% to 96% vs. NPS/OPS: 93% to 98%). When comparing saliva with NPS as a reference standard, sensitivity values ranged from 60% to 96%, with the majority of the studies showing a mean sensitivity of 85%. The specificity values settled over 90% in the majority of cases. However, the “false-positive” subjects were often symptomatic patients with clinical and/or radiological signs of COVID-19 and a negative NPS. It is ascertained that the nasopharyngeal swab is associated with a false-negative rate of approximately 30% after the onset of symptoms (Kucirka et al. 2020), and this feature could have introduced a misclassification bias in the diagnostic accuracy of salivary analysis. Within this framework, a concordance analysis between the 2 samples (k Cohen statistics) is more appropriate to verify the utility of saliva in the molecular diagnostic workflow, and further studies should consider this issue.

Most studies on the detection of viral RNA through saliva have been conducted by recruiting COVID-19 patients or individuals with suspicious symptoms, while only few studies recruited cohorts of asymptomatic patients. The results related to this group were discordant. Although some studies reported a lower sensitivity of saliva in a group of asymptomatic individuals (Caulley et al. 2020; Nagura-Ikeda et al. 2020), other researchers, on the contrary, have highlighted the clinical utility of this oral fluid in detecting SARS-CoV-2 in this population group (Chau et al. 2020; Migueres et al. 2020). For instance, a recent study identified asymptomatic carriers among NPS-negative healthcare workers just through saliva (Wyllie et al. 2020). Thus, it can be concluded that salivary rRT-PCR provides relevant, reliable data that can be used in addition to the reference standard (i.e., NPS) to detect false-negative cases by respiratory swab analysis, thereby increasing the overall sensitivity of standard molecular-based testing (Hanson et al. 2020).

With respect to direct rRT-PCR, 2 groups tested this more rapid procedure on salivary samples and noted a sensitivity that was only slightly lower than the sensitivity shown by the standard protocol with RNA extraction (Nagura-Ikeda et al. 2020) or even superimposable (Fukumoto et al. 2020). These findings demonstrate that the presence of RNases in saliva does not impair such an alternate protocol, which bypasses the classic RNA isolation and purification to reduce the risk of human error during this phase.

Finally, the time required for performing this procedure ranged between 30 and 60 min, ensuring more rapid diagnosis (Chen et al. 2020).

## Point-of-Care Technology for Salivary Diagnosis of COVID-19

Point-of-care testing (POCT) is a medical diagnostic test performed at the time and place of patient care and assistance, that is, the medical office or screening checkpoint. This procedure does not require a centralized laboratory setting, avoiding thus overcrowding and expensive transport media, and it usually provides results within 30 to 60 min.

### Reverse Transcription Loop-Mediated Isothermal Amplification

The reverse transcription loop-mediated isothermal amplification (RT-LAMP) technique has attracted attention for the diagnosis of several infectious diseases during the past decade, such as those caused by the Ebola and Zika viruses (Sabalza et al. 2018). RT-LAMP is a 1-step nucleic acid amplification method that is used to diagnose infectious diseases caused by bacteria or viruses. The commonly used PCR method described above relies on thermal cycling (i.e., cycles of heating and cooling) to facilitate DNA double-helix denaturation and amplification. In contrast, RT-LAMP does not require these cycles and is performed at a constant temperature between 60°C and 65°C. Similar to RT-PCR, RT-LAMP is preceded by reverse transcription for the synthesis of cDNA from RNA sequences. Subsequently, cDNA is amplified using DNA polymerase. Therefore, RT-LAMP is very effective in detecting viruses with an RNA genome.

Several groups in the world have been studying the possibility of applying RT-LAMP technology in combination with a colorimetric qualitative analysis to realize a point-of-care technology to be used in medical practice or in low-income countries, which suffer from a lack of a centralized laboratory network facilities. This technology has also been tested on salivary samples collected from COVID-19 patients without an RNA extraction step. Results were available after 30 min and assessed on the basis of the sample color change when the viral RNA was present (Lalli et al. 2020).

Salivary RT-LAMP offers several advantages for point-of-care diagnostic challenges. First, the salivary sample is self-collected by the patient and does not require RNA extraction. Second, it provides easily interpretable results within 1 h, and it does not require any laboratory devices or complex technologies, apart from a heat block (Lamb et al. 2020; Wei et al. 2020).

For example, EasyCOV (SkiCell and Sys2Diag/CNRS) is a colorimetric RT-LAMP assay designed for salivary analysis. The results can be read by observing the color of the sample inside the test tube. A color change from orange to yellow indicates that the sample is positive and SARS-CoV-2 is present (L’Helgouach et al. 2020).

#### Diagnostic Performance of Salivary RT-LAMP Assay

The sensitivity of RT-LAMP for SARS-CoV-2 using upper and lower respiratory tract specimens has been reported to be equivalent to that of rRT-PCR, showing a 95% agreement with rRT-PCR (Lamb et al. 2020). However, 1 study highlighted that the sensitivity of RT-LAMP in detecting SARS-CoV-2 was lower than that of the classic rRT-PCR test for COVID-19 in saliva specimens (RT-LAMP: 70.9% vs. rRT-PCR: 81.6%); thus, more studies are needed (Nagura-Ikeda et al. 2020).

### Other Point-of-Care Technologies under Development

Other groups are developing new technological solutions for point-of-care molecular-based diagnostics. Specific High-sensitivity Enzymatic Reporter UnLOCKing (SHERLOCK) technology combines viral RNA amplification with LAMP and Clustered Regularly Interspaced Short Palindromic Repeat (CRISPR)–mediated detection. This procedure (STOPCovid) is simple to perform, and the results can be visualized with lateral flow strips in a point-of-care setting. A preliminary report showed a successful diagnosis in 12 positive and 5 negative COVID-19 patients (Joung et al. 2020). The test returns results in 40 to 70 min.

DNA nanoscaffold hybrid chain reaction (DNHCR)–based nucleic acid assay strategy is an innovative technology that can provide results for salivary specimens within 10 min (Jiao et al. 2020). Single-strand recombinase polymerase amplification (ssRPA) allows rapid amplification of double-stranded DNA (dsDNA), conversion to single-stranded DNA (ssDNA), and sequence-specific, hybridization-based readout with a lateral flow dipstick. Initial experimentation of the proof of concept seems to be associated with a very high sensitivity in viral detection (Kim, Yaseen, et al. 2020). Finally, Raman spectroscopy, a technology that is based on the principle of inelastic scattering of light, has been tested for the detection of SARS-CoV-2 viral RNA after it yielded interesting findings for other viral infections (92.5% sensitivity and 88.8% specificity) (Desai et al. 2020).

## Antibody Testing in the Saliva

Active infection can be detected by molecular-based testing for viral RNA, but this approach cannot be used for seroprevalence investigations. Antibody testing on blood samples (or saliva) is useful for determining historic exposure to the virus and may provide insight into the immunological status of the individual (Faustini et al. 2020). It can be performed both by lateral flow assay (LFA) directly in the field or by enzyme-linked immunosorbent assay (ELISA) and/or chemiluminiscent assay technologies in a centralized laboratory.

### Results of Salivary Antibody Testing

Only a few reports have investigated saliva as a specimen to detect antibodies directed against SARS-CoV-2. In one of these studies, antispike (but not nucleocapsid) IgG, IgA, and IgM antibody responses were readily detectable in saliva from nonhospitalized symptomatic and asymptomatic patients. Interestingly, antibody responses in the saliva and serum and symptoms are largely independent of one another (Faustini et al. 2020). In contrast to these results, another study evaluated the results of a multiplex immunoassay to detect specific antibodies in the crevicular fluid and found that SARS-CoV-2 antigen-specific IgG responses in matched serum and saliva samples were significantly correlated. The kinetics of IgG, IgA, and IgM in the saliva were consistent with those observed in serum (Randad et al. 2020).

One advantage of saliva over blood samples is the presence of IgA antibodies. Serum IgAs have been detected in the serum of COVID-19 patients and appear to be detectable earlier than IgM or IgG antibodies, possibly as early as 2 d after the onset of symptoms (Yu et al. 2020). In contrast to IgM and IgG antibodies, which are usually less concentrated in saliva than in blood, IgAs are well represented because they are the main antibody class found in mucosal secretions.

Recently, a point-of-care ELISA test protocol specifically designed for IgA detection in saliva (Brevitest IgA Salivary Mucosal Test [BRAVO]) reported a positive predictive agreement of 92% and a negative predictive agreement of 97% in a group of 38 patients who had previously tested PCR positive (Varadhachary et al. 2020).

## Rapid Salivary Antigen Tests and Point-of-Need Devices

Point-of-need testing (PONT) refers to the use of diagnostic tests outside the medical offices or laboratories, where a very rapid diagnosis is required to screen the population, such as cinemas, theaters, schools, universities, sport facilities, restaurants, shopping centers, and airports (Sabino-Silva et al. 2020). It usually does not require medical personnel or special equipment and is typically performed with a simple device that can be easily used by everyone. One example is the pregnancy test.

A rapid antigen test is a rapid diagnostic test that detects the presence of an antigen (i.e., a viral protein on the surface). This distinguishes it from other medical tests that detect antibodies (antibody tests) or nucleic acids (molecular-based tests). Unlike serological tests, an antigen test cannot release a presumed immune passport, since it does not identify the presence of specific IgG and/or IgM antibodies against SARS-CoV-2. It simply detects the presence of the virus directly at the moment of analysis. This feature accounts for its suitability in a mass screening program during the postepidemic phase.

To achieve this aim, any antigen test needs to be capable of widespread delivery in the targeted territory, in addition to being easily manageable by nonmedical health care personnel and having a fair price.

**Figure 4.**
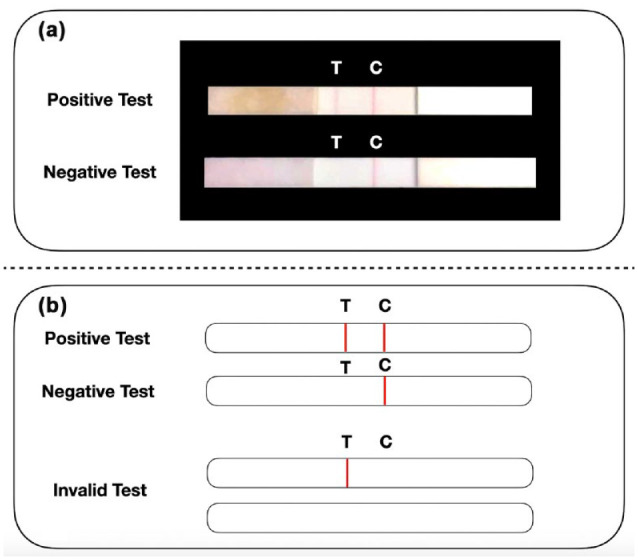
Rapid salivary test (RST) based on lateral flow assay (LFA). (**a**) The rapid antigen test recognizes the presence of a specific viral antigen, such as the spike protein. Briefly, the salivary sample is applied to a sample pad diluted with a specific buffer, where it runs along the nitrocellulose membrane reaching the absorbent pad placed at the opposite site of the strip. (**b**) When both the “test-line” (T-line) and the “control line” (C-line) are visible, the test is “positive” (Severe Acute Respiratory Syndrome Coronavirus 2 is present). When only the C-line is visible, the test is “negative.” The test is “invalid” when the C-line is invisible, regardless of the presence of the T-line. This picture represents the proof of concept and the prototype of the diagnostic test published by our group (Azzi, Baj, et al. 2020).

Keeping these priorities in mind, we have recently published the results of a study dealing with the diagnostic accuracy of a Rapid Salivary Test (RST) based on the LFA to detect SARS-CoV-2 (Azzi, Baj, et al. 2020). The test provided results in less than 10 min, detecting the presence of the spike protein in the salivary sample. Briefly, the saliva collected from the subject is applied to a sample pad, and it runs along a nitrocellulose membrane by capillarity. After 5 to 10 min, the result can be read: if 2 colored bands are visible (both test and control lines), the subject is infected, while if only the control line is visible, the subject is not infected (Fig. 4). We reported a high sensitivity (93%), in contrast to other studies, which reported a low sensitivity (Nagura-Ikeda et al. 2020). These differences are probably due to the different performances of the antibodies used.

The possibility of a rapid antigen test based on salivary diagnosis has received increasing attention over the past few months, and the prospect of developing more technologically advanced diagnostic systems using smartphone-based microfluidic systems with specific biosensors represents one of the greatest challenges for the near future, especially in case for other pandemic outbreaks (Farshidfar and Hamedani 2020). An Israeli group of researchers recently announced a salivary test that could detect the presence of the virus in 1 s with a 95% success rate by using a small spectral device and artificial intelligence (SpectraLIT™).

## Conclusion

The role of salivary diagnosis during the COVID-19 pandemic has received increasing attention from researchers worldwide for several reasons. First, the sensitivity of the salivary sample is comparable to that of respiratory samples. Second, the oral fluid is self-collected by the subjects who are going to be tested; thus, the risk of viral transmission for health care workers is dramatically reduced. Third, saliva can be easily managed since its collection does not require specialized health care personnel and can be also performed by trained non–health care professionals. Finally, the use of this technique can spare medical human resources during the peak of a pandemic outbreak, which is of paramount importance for the national health system of a country dealing with such an event.

**Figure 5.**
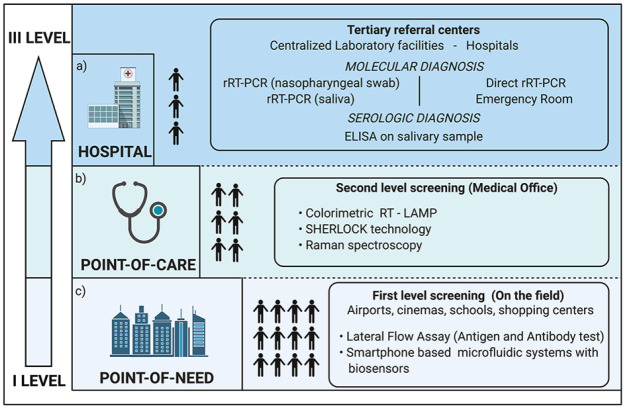
The suitable setting for each salivary diagnostic procedure. (**a**) In a hospital or centralized laboratory facility, real-time reverse transcription polymerase chain reaction (rRT-PCR) represents the reference standard. However, this procedure requires an adequate supply of reagents and the presence of specialized personnel. Only selected cases should undergo this procedure, avoiding thus the crowding of laboratories and other health facilities. Direct rRT-PCR without RNA extraction can be used as a preliminary analysis to screen the suspected COVID-19 patients with suggestive symptoms when entering the emergency room. (**b**) Point-of-care technology represents a valid and useful tool to help physicians who treat patients in a setting outside the hospital, such as a general practitioner’s surgery. (**c**) Point-of-need devices are suitable for mass screening programs and for the analysis of salivary samples directly on the field where the test is needed, like a school, a cinema or theater, a restaurant, or an airport.

However, not all diagnostic tests are suitable for the diagnosis in every setting (Fig. 5). Although rRT-PCR represents the reference standard for molecular diagnosis in salivary samples, the time required for the analysis limits its application in a mass screening program; thus, it should be regarded as the preferred test in hospitals, suitable for COVID-19 inpatients or for confirming the positive diagnosis provided by tests on other samples, especially in cases yielding suspected false-negative results by nasopharyngeal swab analysis. Direct PCR assays without RNA extraction could be easily applied in an emergency room, in which the operators need certain results quickly, reducing the risk of personnel contamination.

Salivary diagnostics find its main field of application in a setting outside the hospital, especially in medical practice (point-of-care). In this context, this kind of technology should provide results within 30 to 60 min and be performed by nonspecialized medical staff, and the devices should be easy to use and portable.

Finally, the role of salivary diagnostics is promising for direct testing in the field (point-of-need) in places of social aggregation. Identifying asymptomatic infectious subjects before they enter an enclosed space and spread the infection to other individuals represents the main worrisome issue for all public institutions, private businesses, or social activities (Lavezzo et al. 2020). The economic crisis that has followed the health emergency caused by the epidemic will soon make it unsustainable to lengthen any widespread lockdown protocol or extend heavy restrictions for people’s travels. Therefore, a mass screening program is necessary, and it should rely on devices that can also be used by nonmedical staff to quickly assess whether an individual is infectious. Rapid salivary antigen tests may represent a key strategy for mass containment of the pandemic outbreak.

## Author Contributions

L. Azzi, contributed to conception, design, and data acquisition, drafted the manuscript; V. Maurino, M. Lualdi, contributed to data acquisition and interpretation, drafted the manuscript; A. Baj, contributed to conception and design, drafted the manuscript; M. Dani, A. d’Aiuto, contributed to data analysis and interpretation, drafted the manuscript; M. Fasano, F. Sessa, contributed to conception, design, critically revised the manuscript; T. Alberio, contributed to conception, design, data analysis, and interpretation, critically revised the manuscript. All authors gave final approval and agree to be accountable for all aspects of the work.

## Supplemental Material

DS_10.1177_0022034520969670 – Supplemental material for Diagnostic Salivary Tests for SARS-CoV-2 Click here for additional data file. Supplemental material, DS_10.1177_0022034520969670 for Diagnostic Salivary Tests for SARS-CoV-2 by L. Azzi, V. Maurino, A. Baj, M. Dani, A. d’Aiuto, M. Fasano, M. Lualdi, F. Sessa and T. Alberio in Journal of Dental Research
