# Supplementary material for: Diagnostic Salivary Tests for SARS-CoV-2
Source: J Dent Res. 2020 Oct 31;100(2):115–23. doi: 10.1177/0022034520969670 (PMC7604673; doi:10.1177/0022034520969670)
Supplement: DS_10.1177_0022034520969670 – Supplemental material for Diagnostic Salivary Tests for SARS-CoV-2 [file DS_10.1177_0022034520969670.pdf]

APPENDIX

VIRAL LOAD IN SALIVARY SAMPLES

In the first report the viral load was determined by relative quantification using rRT-PCR. Our group found that *cycle threshold* (*Ct*, the number of cycles required for the fluorescent signal to exceed background level) values were correlated with lactate dehydrogenase (LDH) values, as evaluated by blood chemistry tests. This finding suggested that the viral load could be linked to disease severity and lung tissue damage (Azzi et al., 2020). Williams and colleagues found that the Ct values recorded in the salivary samples were higher than the NPS Ct values, probably indicating a higher viral load in the respiratory samples (Williams et al. 2020). Other studies did not report significant differences between saliva and respiratory samples with respect to Ct values (Iwasaki et al., 2020; Kim et al., 2020).

The first quantitative analysis with an absolute quantification method based on an internal standard of the viral load in the saliva was carried out by To and colleagues on the posterior oropharyngeal saliva (To et al., 2020). The authors found that the viral load was higher during the first week after the onset of symptoms, and it gradually decreased subsequently. Other studies have confirmed these results (Zheng et al., 2020; Zhu et al., 2020). These findings are in contrast to those previously reported during the MERS-CoV and SARS-CoV outbreaks, in which the salivary load appeared to be higher during the second week. The correlation between the salivary viral load and the degree of disease severity is debatable, with several studies reporting that the salivary load shows no direct correlation with a worse clinical progression (To et al., 2020; Zhu et al. 2020).

Another feature that has been highlighted is the lower temporal variability in the saliva than in the NPS (Wyllie et al., 2020). Several case reports have been published about people who had a

negative NPS and then tested positive with a new swab. In these cases, saliva seems to maintain positivity more consistently (Wölfel et al., 2020).

Only a few reports have considered the salivary viral load in asymptomatic subjects (Chau et al., 2020; Willye et al., 2020). Data from these studies suggest that asymptomatic subjects show a viral load similar to that detected in symptomatic subjects, but it is lower in comparison with the NPS, and it is associated with faster viral clearance. However, even when the salivary load was lower, clusters of transmission were described (Chau et al. 2020). Other studies reported higher viral loads in saliva than in oropharyngeal swab (Bosworth et al., 2020). These findings, together with the early detection of high viral loads in infected patients, explain the necessity of a serious discussion about the role played by asymptomatic carriers in the transmission of COVID-19 infection, a role that seems to be more relevant in this pandemic than in the 2003 SARS outbreak (Lavezzo et al. 2020).

REFERENCES

Azzi L, Carcano G, Gianfagna F, Grossi PA, Dalla Gasperina D, Genoni A, Fasano M, Sessa F, Tettamanti L, Carinci F, et al. 2020c. Saliva is a reliable tool to detect SARS-CoV-2. *J Infect* 81(1):e45-e50

Bosworth A, Whalley C, Poxon C, Wanigasooriya K, Pickles O, Aldera EL, Papakonstantinou D, Morley GL, Walker EM, Zielinska AE, et al. 2020. Rapid implementation and validation of a cold-chain free SARS-CoV-2 diagnostic testing workflow to support surge capacity. *J Clin Virol* 128:104469 doi: 10.1016/j.jcv.2020.104469

Chau NVV, Thanh Lam V, Thanh Dung N, Yen LM, Minh NNQ, Hung LM, Ngoc NM, Dung NT, Man DNH, Nguyet LA, et al. 2020. The natural history and transmission potential of asymptomatic SARS-CoV-2 infection. *Clin Infect Dis* doi: 10.1093/cid/ciaa711 [Online ahead of print]

Iwasaki S, Fujisawa S, Nakakubo S, Kamada K, Yamashita Y, Fukumoto T, Sato K, Oguri S, Taki K, Senjo H, et al. 2020. Comparison of SARS-CoV-2 detection in nasopharyngeal swab and saliva. *J Infect* doi: 10.1016/j.jinf.2020.05.071 [Online ahead of print]

Lavezzo E, Franchin E, Ciavarella C, Cuomo-Dannenburg G, Barzon L, Del Vecchio C, Rossi L, Manganelli R, Loregian A, Navarin N, et al. 2020. Suppression of a SARS-CoV-2 outbreak in the Italian municipality of Vo'. *Nature* doi: 10.1038/s41586-020-2488-1 [Online ahead of print]

Kim SE, Lee JY, Lee A, Kim S, Park KH, Jung SI, Kang SJ, Oh TH, Kim UJ, Lee SY, et al. 2020. Viral load kinetics of SARS-CoV-2 infection in saliva in Korean patients: a prospective multi-center comparative study. *J Korean Med Sci* 35(31):e287

To KK, Tsang OT, Leung WS, Tam AR, Wu TC, Lung DC, Yip CC, Cai JP, Chan JM, Chik TS, et al. 2020. Temporal profiles of viral load in posterior oropharyngeal saliva samples and serum antibody responses during infection by SARS-CoV-2: an observational cohort study. *Lancet Infect Dis* 20(5):565-574

Williams E, Bond K, Zhang B, Putland M, Williamson DA. 2020. Saliva as a non-invasive specimen for detection of SARS-CoV-2. *J Clin Microbiol* doi: 10.1128/JCM.0076-20 [Online ahead of print]

Wölfel R, Corman VM, Guggemos W, Seilmaier M, Zange S, Müller MA, Niemeyer D, Jones TC, Vollmar P, Rothe C, et al. 2020. Virological assessment of hospitalized patients with COVID-2019. *Nature* 581(7809):465-469

Wyllie AL, Fournier J, Casanovas-Massana A, Campbell M, Tokuyama M, Vijayakumar P, Warren JL, Geng B, Muenker MC, Moore AJ, et al. 2020. Saliva or nasopharyngeal swab specimens for detection of SARS-CoV-2. N Engl J Med doi: 10.1056/ NEJMc2016359 [Online ahead of print]

Zheng S, Fan J, Yu F, Feng B, Lou B, Zou Q, Xie G, Lin S, Wang R, Yang X, et al. 2020. Viral load dynamics and disease severity in patients infected with SARS-CoV-2 in Zhejiang province, China, January-March 2020: retrospective cohort study. *BMJ* 369:m1443

Zhu J, Guo J, Xu Y, Chen X. 2020. Viral dynamics of SARS-CoV-2 in saliva from infected patients. *J Infect* doi: 10.1016/j.jinf.2020.06.059 [Online ahead of print]
